# A comparison of high-throughput plasma NMR protocols for comparative untargeted metabolomics

**DOI:** 10.1007/s11306-020-01686-y

**Published:** 2020-05-01

**Authors:** Nikolaos G. Bliziotis, Udo F. H. Engelke, Ruud L. E. G. Aspers, Jasper Engel, Jaap Deinum, Henri J. L. M. Timmers, Ron A. Wevers, Leo A. J. Kluijtmans

**Affiliations:** 1 Translational Metabolic Laboratory, Department of Laboratory Medicine, Radboudumc, Geert Grooteplein Zuid 10, 6525 GA Nijmegen, The Netherlands; 2 Institute for Molecules and Materials, Radboud University, Houtlaan 4, 6525 XZ Nijmegen, The Netherlands; 3 Present Address: Biometris, Wageningen UR, Droevendaalsesteeg 1, 6708 PB Wageningen, The Netherlands; 4 Department of Internal Medicine, Radboudumc, Geert Grooteplein Zuid 10, 6525 GA Nijmegen, The Netherlands

**Keywords:** NMR, Metabolomics, High-throughput, Large scale, Classification, LED

## Abstract

**Introduction:**

When analyzing the human plasma metabolome with Nuclear Magnetic Resonance (NMR) spectroscopy, the Carr–Purcell–Meiboom–Gill (CPMG) experiment is commonly employed for large studies. However, this process can lead to compromised statistical analyses due to residual macromolecule signals. In addition, the utilization of Trimethylsilylpropanoic acid (TSP) as an internal standard often leads to quantification issues, and binning, as a spectral summarization step, can result in features not clearly assignable to metabolites.

**Objectives:**

Our aim was to establish a new complete protocol for large plasma cohorts collected with the purpose of describing the comparative metabolic profile of groups of samples.

**Methods:**

We compared the conventional CPMG approach to a novel procedure that involves diffusion NMR, using the Longitudinal Eddy-Current Delay (LED) experiment, maleic acid (MA) as the quantification reference and peak picking for spectral reduction. This comparison was carried out using the ultrafiltration method as a gold standard in a simple sample classification experiment, with Partial Least Squares–Discriminant Analysis (PLS-DA) and the resulting metabolic signatures for multivariate data analysis. In addition, the quantification capabilities of the method were evaluated.

**Results:**

We found that the LED method applied was able to detect more metabolites than CPMG and suppress macromolecule signals more efficiently. The complete protocol was able to yield PLS-DA models with enhanced classification accuracy as well as a more reliable set of important features than the conventional CPMG approach. Assessment of the quantitative capabilities of the method resulted in good linearity, recovery and agreement with an established amino acid assay for the majority of the metabolites tested. Regarding repeatability, ~ 85% of all peaks had an adequately low coefficient of variation (< 30%) in replicate samples.

**Conclusion:**

Overall, our comparison yielded a high-throughput untargeted plasma NMR protocol for optimized data acquisition and processing that is expected to be a valuable contribution in the field of metabolic biomarker discovery.

**Electronic supplementary material:**

The online version of this article (10.1007/s11306-020-01686-y) contains supplementary material, which is available to authorized users.

## Introduction

Human metabolome profiling can lead to a better understanding of disease mechanisms and to potential biomarkers for improved diagnosis (Jansson et al. 2009; Malatji et al. 2017; Sharma et al. 2013; Van Karnebeek et al. 2016). Although there is a wide variety of biomaterial for metabolomics experts to choose from, human blood’s homeostatic ability, easy collection and the fact that it flows through every human organ, render it a particularly attractive option for investigating disease-associated pathways (Nagana Gowda et al. 2015).

Proton nuclear magnetic resonance (^1^H-NMR) spectroscopy was the technology that kicked off the field and is one of the two main methods used. The technique has inherent quantitative capabilities (Nagana Gowda et al. 2018), high reproducibility, requires minimal sample preparation, is non-destructive for samples and offers the possibility for compound identification by structure elucidation, all essential characteristics for biomarker discovery (Gowda et al. 2008). Although NMR spectroscopy is limited by low sensitivity compared to mass spectrometry (Gowda et al. 2008) as well as signal overlap in spectra, it continues to find widespread applications (Cheng et al. 2019; Mora-Ortiz et al. 2019; Turkoglu et al. 2019; Yousf et al. 2019). When applied to plasma, a number of considerations should be made in regard to its performance in metabolome profiling with respect to optimal sample group separation that can be explained by a solid biochemical background.

The first of these considerations is related to the presence of proteins and lipids in plasma samples. These macromolecules give rise to numerous signals that overlap with those originating from small polar metabolites. Hence, an appropriate method for polar metabolomics should be able to separate relevant signals from these confounders. Typically, when analyzing plasma with NMR spectroscopy, macromolecules are analyzed using the Longitudinal Eddy-Current Delay (LED) (Beckonert et al. 2007), whereas low molecular weight compounds by means of precipitation (Nagana Gowda et al. 2015; Nagana Gowda and Raftery 2014), ultrafiltration (Wevers et al. 1994) or Carr–Purcell–Meiboom–Gill (CPMG) (Beckonert et al. 2007). Ultrafiltration is an approach that requires the use of filters to remove macromolecules and is capable of quantifying a larger number of metabolites than CPMG. Ultrafiltration has been used to discover inborn errors of metabolism (Engelke et al. 2008) and to profile the human serum metabolome (Psychogios et al. 2011). In the latter study, the approach led to the absolute quantification of 49 metabolites, a high end point number of NMR-quantifiable human serum metabolites. The main limitations of this gold standard include laboriousness and cost (Wallmeier et al. 2017) due to the tedious filtration step that requires the use of expensive filters, which hamper the method’s applicability in large scale studies. An alternative would be protein precipitation by means of the methanol protocol (Nagana Gowda and Raftery 2014). This approach, however, retains small residual macromolecules and is aimed at targeted NMR. Instead, pulse programs that suppress unwanted compound signals (Beckonert et al. 2007; de Graaf et al. 2015; de Graaf and Behar 2003; Liu et al. 2002; Wallmeier et al. 2017) are more commonly used in untargeted NMR studies of large cohorts. The CPMG pulse sequence has seen extensive use in the field of plasma 1D NMR metabolomics, but is limited by the number of detectable metabolites, signal attenuation, baseline distortion due to residual macromolecule signals, low resolution and limited quantification accuracy (Nagana Gowda et al. 2015). As for the LED method, it is possible to focus on small metabolites instead of large ones by recording high gradient diffusion spectra and subtracting them from their respective low gradient counterparts. This approach has been found to correlate well with results obtained from ultrafiltration (de Graaf et al. 2015; de Graaf and Behar 2003), but has not yet been evaluated in terms of sample classification, or quality of the obtained metabolite signature.

To follow up spectral data collection, data reduction is key for statistical analysis methods. Equidistant bucketing or binning is a well-known data reduction approach, where each spectrum is split in a predefined number of integral bins and the result is directly used for statistics (Bharti and Roy 2012), but has certain well-known limitations (Beirnaert et al. 2018; Vu and Laukens 2013). Nevertheless, binning continues to know use today (Castiglione Morelli et al. 2019; Hanifa et al. 2019; Jiang et al. 2019; Singh et al. 2019). A relatively new alternative involves peak picking and grouping by using wavelets (Beirnaert et al. 2018). Regarding quantification standards, Trimethylsilylpropanoic acid (TSP) is the compound of choice for aqueous solutions. However, given its tendency to bind to protein (Wallmeier et al. 2017), this compound is not suitable for unfiltered samples. An option mostly preferred for quantitative NMR (qNMR) is maleic acid (Salem and Mossa 2012), but to date has not been utilized in metabolic studies.

We compare here three untargeted plasma NMR metabolomics and two spectral processing methods. Replicate samples representing two forms of endocrine hypertension, primary aldosteronism (PA) as well as pheochromocytoma and paraganglioma (PPGL) were analyzed using each method and discriminated using supervised multivariate statistics. The results from models separating PA from PPGL were used for the comparison of the methods’ performances in group separation. In addition, we assess the performance of the optimal approach in metabolite quantification using maleic acid as the internal quantification standard. Based on the results presented herein, the novel plasma NMR metabolomics procedure developed is expected to yield valuable sample stratification models, as well as metabolic signatures for understanding the underlying biochemical differences involved in the comparison under investigation.

## Materials and methods

### Chemicals and standards

Di-Sodium hydrogen phosphate dihydrate (Na_2_HPO_4_⋅2H_2_O) and Sodium azide (NaN_3_) were purchased from Merck, D_2_O 99% and MA from Sigma-Aldrich. Standards used (Phenylalanine, Methionine, Lysine, Threonine, Creatine and Hypoxanthine) were obtained from Sigma-Aldrich, except for Glycine which was obtained from Scharlab, Alanine from Fluka and Creatinine from BioChemika. Deionized water was prepared using a Milli-Q Advantage A10 Water Purification System from Merck.

In order to keep pH stable and chemical shift variability to a minimum, a buffer solution consisted of 0.43 mM MA, 2.18 mM NaN_3_ and 2 mM Na_2_HPO_4_ was prepared. The buffer’s pH was adjusted to 7.4 using HCl and NaOH solutions. This solution was made for samples that were prepared as described below, according to the protocol proposed by Bernini et al. (2011). A separate phosphate buffer solution, containing both TSP and MA as internal standards was prepared for an initial experiment to compare MA to TSP signal line broadening in replicate plasma samples. This solution consisted of 3.32 mM TSP, 18.77 mM MA, 2.2 mM NaN_3_ and 142.07 mM Na_2_HPO_4_, whereas its pH was adjusted to 7.4 using HCl and NaOH solutions.

The master stock solution containing all selected compounds was prepared by dissolution of 5.54 mM Glycine, 2.38 mM Alanine, 4.77 mM Phenylalanine, 0.56 mM Methionine, 3.17 mM Lysine, 1.14 mM Threonine, 1.07 mM Creatine, 0.99 mM Creatinine and 0.29 mM Hypoxanthine in 25 mL dH_2_O. Out of this master stock solution, subsequent dilutions of 3 ×, 5 × and 10 × were prepared. These standard stock solutions were prepared thusly to cover as much concentration range as is biologically relevant for each compound.

### Samples

**Fig. 1:**
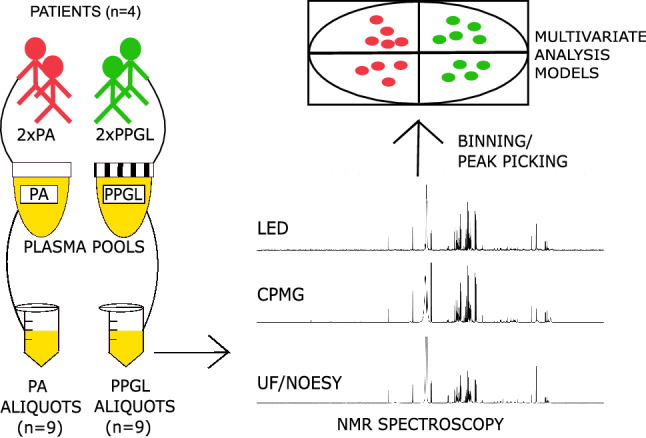
Sample preparation and subsequent actions for the comparison of the three methods, applied as described in the methods section. Plasma collected from the two “PA” patients was pooled and aliquoted 9 times to create the replicate samples of the “PA” group. The process was repeated for the PPGL group. All 18 samples underwent analysis by NMR using ultrafiltration and the NOESY pulse sequence, CPMG and the LED process. Spectra were converted to data tables through the processes of binning and peak picking. Finally, Multivariate Analysis models were employed to investigate differences in the signatures obtained from each method

For all experiments carried out, four sets of plasma samples were prepared. For the proof of principle application, as shown in Fig. 1, a total of 18 plasma samples were collected from heparin blood originating anonymously from four patients; two patients suffered from pheochromocytoma/paraganglioma (PPGL) and two from primary aldosteronism (PA). All four patients provided informed consent. After collection in heparin tubes, the blood was centrifuged for 10 min at 3000×*g* to separate the plasma from cells. Plasma was pooled per disease and aliquoted. Finally, 9 PPGL and 9 PA replicates were prepared and stored at − 80 °C until analysis.

A number of replicate samples intended for internal standard comparison were prepared. A volume of 8 mL of plasma pooled from a set of anonymized patient samples used for clinical studies was aliquoted 8 times (350 μL each) to compare MA to TSP. Subsequently, four pooled plasma aliquots were subjected to ultrafiltration and the remaining four to unfiltered sample preparation for NOESY 1D NMR.

A set of 435 QC samples were prepared by pooling ~ 450 mL of plasma obtained from 390 anonymized plasma samples. This large volume of plasma was aliquoted, resulting in 1 mL for each QC sample that was subsequently stored at − 80 °C and used for quantification experiments and intra- and inter-batch repeatability assessment of NMR plasma analyses.

For the standard addition experiment, 15 plasma samples were prepared by adding 100 μL from each stock solution to 400 μL of QC plasma. Specifically, 100 μL of stock solutions 1 × , 3 × , 5 × , 10 × and dH_2_O were added separately to sets of three QCs to make up 15 samples. These samples were analyzed by NMR and routine ion exchange chromatography for amino acid analysis (AAA).

### Sample preparation

The ultrafiltration procedure was performed according to manufacturer’s instructions with an additional washing step (Wevers et al. 1994). Filters with a 10 kDa cutoff were obtained from Sigma-Aldrich (Centrisart I Centrifugal Ultrafiltration Unit) and used for macromolecule removal from plasma samples. Briefly, after two cycles of rinsing the filters with 2.5 mL dH_2_O and centrifugation at 2113×*g* for 5 min to remove glycerol, 1 mL from each aliquot was transferred to the filter outer tube. Filters were centrifuged at 2113×*g* for 30 min, resulting in ~ 600 μL of filtrate for each sample. These were stored at − 80 °C until analysis with NMR. Before NMR analysis, both filtered and unfiltered samples were thawed at room temperature. A volume of 350 μL was transferred from each sample to an eppendorf safe-lock tube (1.5 mL) to which the same volume of buffer solution was added. Each safe-lock tube’s contents were mixed using a Retsch vortex mixer and centrifuged at 16,100×*g*, for 5 min. Finally, 650 μL of each sample were pipetted to a NMR tube.

### NMR experiments

^1^H-NMR spectra were recorded at 298 K on a Bruker DRX AVANCE spectrometer equipped with a triple resonance inverse 5 mm probe head operating at 500.13 MHz or 11.74 T of magnetic field strength. ^1^H-NMR experiments (Fig. 1) alternated between the NOESY pulse sequence (64 k data points) with water presaturation for filtered samples and the Carr-Purcell-Meiboom-Gill (CPMG), Longitudinal Encode-Decode (LED) with 2% gradient strength and LED with 70% gradient strength pulse sequences for unfiltered samples (128 k data points for both experiments). The LED pulse sequence had the form -RD-901-G1-1801-G1-901-G2-T-901-G1-1801-G1-901-G2-t-901-acquire FID, where RD is a relaxation delay, 901 is a 901 RF pulse, G1 is the pulsed-field gradient that is applied to allow editing, 1801 is a 1801 RF pulse, G2 is a spoil gradient applied to remove unwanted magnetization components. The diffusion delay D is the time during which the molecules are allowed to diffuse—this is the period (901-G1-1801-G1-901-G2-T-); and t is a delay to allow the longitudinal eddy currents caused within the sample to decay (Beckonert et al. 2007). For CPMG experiments, 300 loop counters were used to sufficiently suppress macromolecule signals, whereas for LED experiments a constant value for receiver gain was preselected. For all experiments, 256 scans were recorded after 8 dummy scans using a relaxation delay of 4 s, 3.25 s acquisition time and 0.3 Hz line broadening. In terms of time, Icon NMR required 5 min for loading each sample and preparation for each experiment, 35 min for each LED and NOESY and 55 min for each CPMG experiment. Spectral resolution was assessed based on the peak width of MA at half height. Both the loop counters for the CPMG and the % gradient strength for the LED experiments were determined based on macromolecule signal suppression, using the 1D NOESY experiment’s results on corresponding filtered samples as reference.

### Quantification assessment

Quantification performance of the method was assessed on the basis of linearity of response (Harmonization 1996), recovery, agreement with amino acid assay (Choudhary and Nagaraja 2007), and repeatability. Targeted metabolite quantification and identification according to MSI guidelines (Sumner et al. 2007) was performed using the Chenomx (Weljie et al. 2006) software (evaluation version 8.4), which is capable of signal deconvolution, necessary for metabolites Glycine, Alanine, Methionine, Threonine, Creatine and Creatinine. For Phenylalanine (doublet at 7.31 ppm) direct peak integration using Topspin version 4.0.6 was employed, as this signal could be used for estimating concentration without the need for deconvolution. Lysine (triplet at 3.01 ppm) was quantified using both methods, as the effect of signal convolution was unclear. Metabolites were quantified using each sample’s levels of formic acid, which were estimated using the internal standard.

Amino acids were quantified using an AminoTac Jeol JLC-500/V amino acid analyzer (Jeol Ltd., Japan), using a commercially available amino acid mixture for calibration and quantification.

### Statistical analysis

**Fig. 2:**
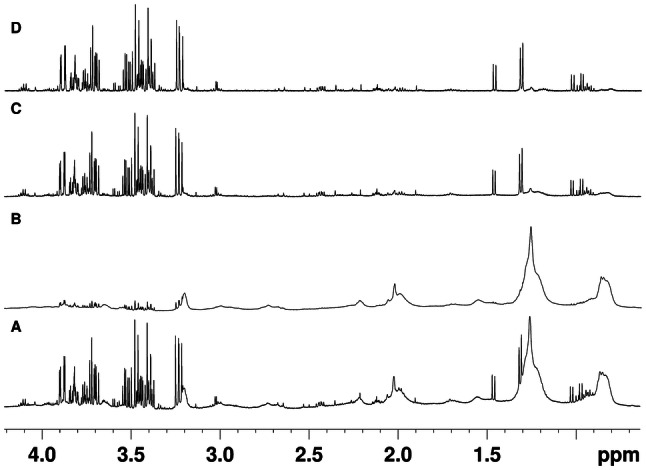
The workflow for obtaining LED spectra, optimized for small molecules. **a** Low gradient strength (2%) LED spectrum of a sample in the PPGL group, **b** high gradient strength (70%) LED spectrum of the same sample, **c** the resulting spectrum from the subtraction of spectrum (b) from spectrum (a) and finally (d) the same spectrum as (c), but with the underground removed using the relevant tool in AMIX v 4.9.2, resulting in a baseline that is not affected by broad macromolecule signals

For spectral processing, all spectra were Fourier transformed, manually phase corrected and the chemical shifts referenced to the glucose doublet at 5.22 ppm, using Bruker Topspin version 3.5. As shown in Fig. 2, to obtain LED spectra the 70% gradient strength LED experiment recorded from each sample was subtracted from its respective 2% LED experiment to retain only small molecule signals, and underground signals were removed with a filter width of 20 Hz using AMIX version 4.9.2 and the area above 10 ppm for noise signal estimation. UF, CPMG and LED spectra were subjected to bucketing as well as peak picking using AMIX and R package “SPEAQ” (Beirnaert et al. 2018), respectively. An alignment step was not utilized, due to blood plasma’s inherent homeostatic ability and the use of a buffer solution, which keep chemical shifts to a minimum. All R packages were loaded on R studio (R studio team 2016) v. 1.1.463 running R (Team 2019) v. 3.4.4. Equidistant bucketing was applied in the area between 0 and 10 ppm of all spectra, by using a bucket width of 0.02 ppm, sum of intensities as an integration method, with option “no scaling” selected. Areas 0.73 to 0.9, 1.09 to 1.30, 1.89 to 1.92, 3.17 to 3.27, 3.35 to 3.54, 3.67 to 3.92, 4.39 to 5.17, 5.23 to 5.37, 5.49 to 5.98, 7.01 to 7.08 and 7.75 to 7.85 ppm, corresponding to macromolecules, water, glucose and urea, 1-methylhistidine (due to chemical shift irregularities) and acetate (due to its presence in the blank in a concentration comparable to samples from one group) were excluded from the bucket table for all subsequent analyses. For peak picking, spectra were initially “read” using the command “readBruker” from the R package “BATMAN” (Hao et al. 2014). The same areas as with the AMIX procedure were excluded. Peak picking, grouping and filling was performed next, using a set of functions in the “SPEAQ” R package. In both the AMIX bucket and SPEAQ peak tables, features not present in at least 80% of samples belonging to either group were removed, as per the recommendations of Southam et al. (2017), for removing any peaks that had too many missing values. Remaining features were scaled to the MA peak/bucket. Next, Probabilistic Quotient Normalization (PQN) (Dieterle et al. 2006) was applied, using all samples to compute the median spectrum ignoring non-detects and missing values were imputed using the K-nearest-neighbor algorithm (KNN) (Armitage et al. 2015; Hrydziuszko and Viant 2012; Southam et al. 2017), as implemented in R package “impute” (Hastie et al. 2019). Finally, the generalized log transformation (GLOG) (Parsons et al. 2007) based on the samples in the PA group (that are technical replicates) is applied using R package “LMGene” (Rocke et al. 2018). AMIX and SPEAQ data were compared using visual inspection of PCA score plots as well as signatures from PLS-DA models. All parameters and scripts can be found on the first author’s GitHub page (https://github.com/NickBliz/High-throughput-Untargeted-Plasma-NMR-Metabolomics_Method) and data can be provided upon request.

Principal Component Analysis (PCA) was used as a method to discover trends and detect outliers, whereas Partial Least Squares Discriminant Analysis (PLS-DA) for separating PA from PPGL samples analyzed with each method. For computing the MVA models, the MixOmics (Rohart et al. 2017) R package was employed. All data were mean-centered. Cross-validation was used to optimize PLS-DA models (Szymańska et al. 2012); each model’s performance in sample classification was computed using the leave-one-out method in order to determine the optimal number (n) of latent variables and subsequently the model was recalculated with these n latent variables, from which the final set of important features was extracted. Quality of supervised models was assessed by double CV (Szymańska et al. 2012); each sample was left out once, the model was recalculated after the optimal number of latent variables was determined by CV and the left out sample was classified as either PA or PPGL. This process was repeated until all samples had been left out once and the sum of all misclassifications was calculated. In all models, variables were detected peaks, whereas observations were analyzed samples.

*Linearity* To assess linearity of the NMR signals, the results from the standard addition experiments were used. The levels of the selected analytes were determined by using MA as the internal standard. R package “gvlma” (Pena and Slate 2019) was used to check whether the linear model assumptions are met as well as package “stats” (Team 2019) to build the linear regression models with mass of metabolite being regressed against concentration found using NMR spectroscopy and Chenomx. To evaluate the quality of the fit, the coefficient of determination (R^2^), the significance (p-value) of the slope coefficient, the Residual Standard Error (RSE) and the prediction error (RSE/average concentration determined by NMR) were all taken into account. A linear model summary is available for every metabolite spiked.

*LOD/LOQ* Limit of detection (LOD) was estimated by multiplying the standard error of y-intercepts of regression lines in the standard addition experiment by 3.3 and dividing by the slope. Limit of quantification (LOQ) was determined by multiplying the LOD by 3.3.

*Recovery* To evaluate recovery, the percentage of difference in mean of three replicate concentrations for six metabolites at four concentration levels in the test solution between the NMR method and AAA was calculated, along with their respective RSD values.

*Agreement with Amino Acid Assay* Bland–Altman comparison plots (based on R package “Bland–Altman–Leh”(Lehnert 2015)) were used to assess the agreement between the two methods for selected metabolites.

*Repeatability* QC samples were analyzed in 45 batches (one QC per batch) using the LED method and the resulting spectra were converted to peaks, which were scaled to each MA peak and normalized using PQN and the median spectrum of all QCs as a reference, to evaluate inter-batch repeatability. For intra-batch repeatability, a set of 19 QC samples were analyzed in one day. Inter- as well as intra-batch RSD values were computed for each peak found in the QC samples.

## Results

### Method comparison and proof-of-principle application

**Fig. 3:**
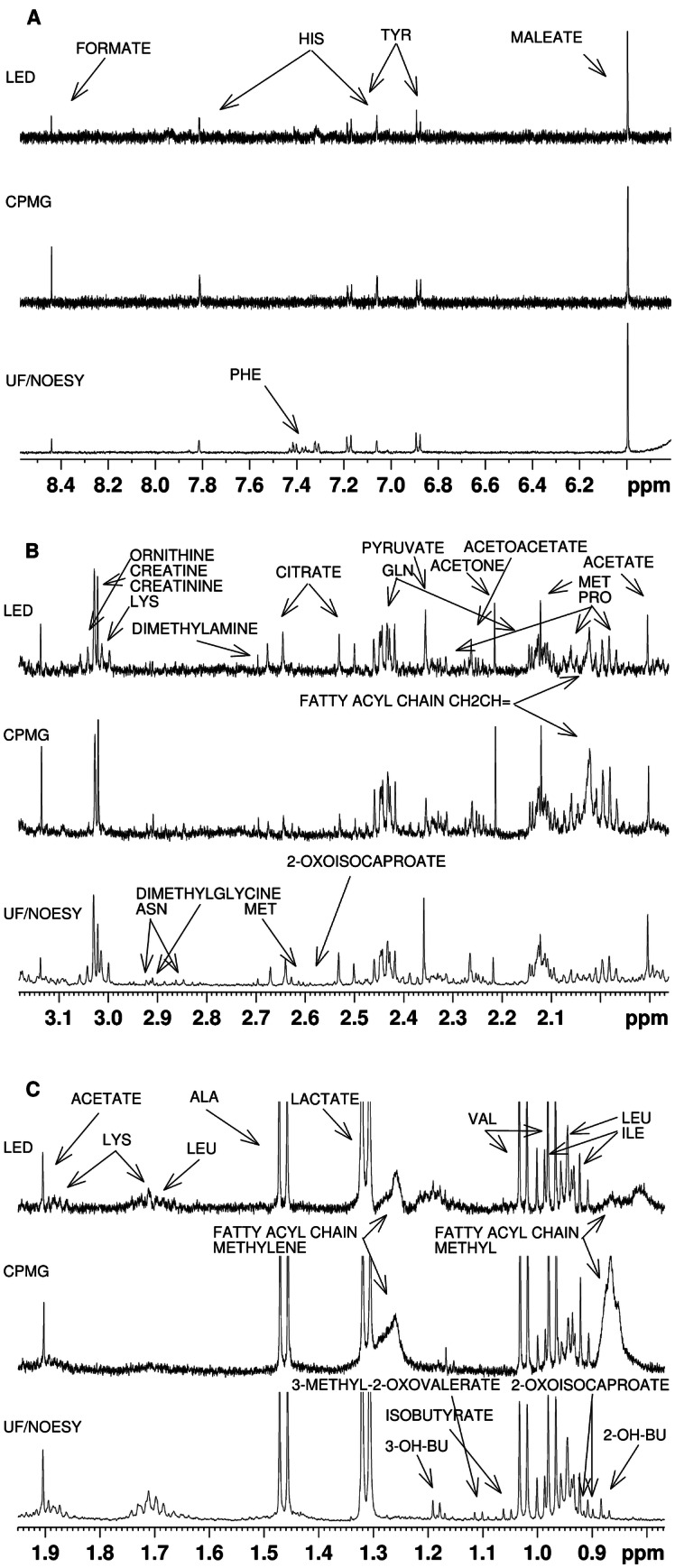
Comparison of the different macromolecular signal suppression NMR methods: diffusion edited (LED), T2-relaxation edited (CPMG), and the ultrafiltration of large molecules NOESY NMR spectrum. **a** The aromatic region, **b** the area from 3.1 to 2 ppm and **c** the area from 0.7 to 1.9 ppm. In each area, the peaks detectable by the LED method are annotated, whereas those only detectable in UF are assigned therein. The most striking differences between methods include the suppression of macromolecular signals and metabolites lysine, ornithine and phenylalanine that are detectable in LED but not in CPMG spectra

The 18 spectra recorded from all methods had a flat baseline and high resolution (Fig. 3), with half height peak width of MA having a median of 0.86 Hz and standard deviation of 0.08 Hz for UF, 0.85 ± 0.09 Hz for CPMG and 0.89 ± 0.11 Hz for LED spectra. To compare signal-to-noise ratios between the spectra shown, the area between 8.50 and 9.99 ppm for noise signal estimation and the maleate peak at 6 ppm were used, resulting in a ratio of 311.09 for the UF spectrum, 74.4 for CPMG and 60.75 for LED. Residual macromolecule signals were observed in unfiltered samples, with their levels being highest in CPMG spectra. Out of all metabolites identified, 36 were detectable by visual inspection in UF spectra, 28 in LED and 26 in CPMG. A list of all detectable metabolites in spectra shown in Fig. 3 can be found in Table 1S of the Supplementary Material.

**Fig. 4:**
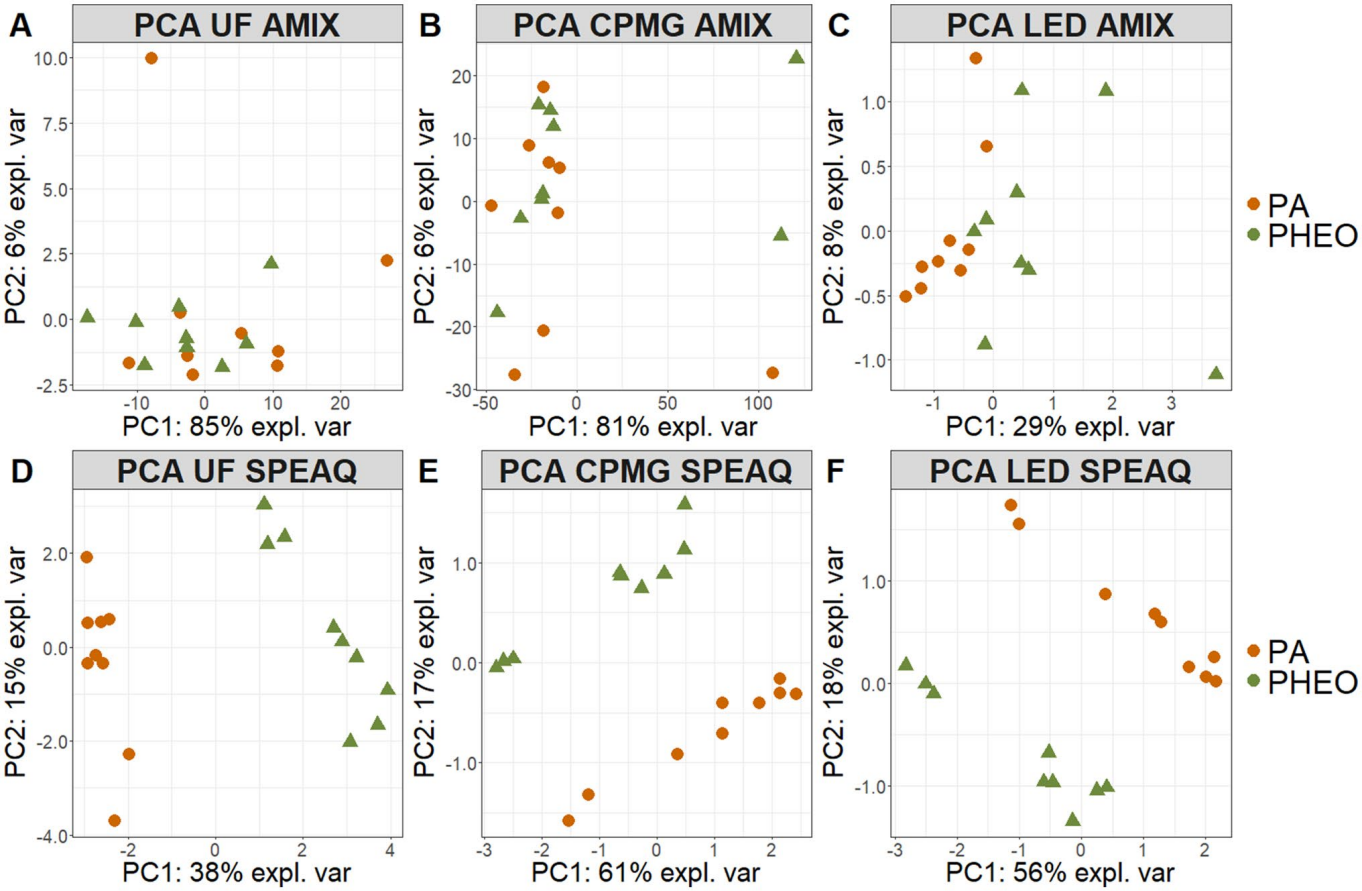
PCA score plots of the first two principal components, obtained after the generalized logarithm was applied as a scaling method on the data collected from each method. **a** UF bucket table, **b** CPMG bucket table, **c** LED bucket table, **d** UF peak table, **e** CPMG peak table and **f** LED peak table. The first principal components mainly explain differences outliers have from the rest of the dataset in AMIX models, but group differences in SPEAQ models

Score plots from PCA models calculated using the AMIX and the SPEAQ data of all methods as shown in Fig. 4, indicated more outliers for the AMIX than for the SPEAQ datasets for all methods, with CPMG resulting in the most. Further, PA samples seem clearly separated from PPGLs according to the first two principal components when using the SPEAQ approach. Group separation was also observed in the 3rd component of the UF AMIX model (not shown) and according to the first two principal components (PCs) of the LED AMIX model (Fig. 4c), albeit to a lesser degree than with the respective SPEAQ-resulting model (Fig. 4f). According to the score plots of the same PCA models, the SPEAQ models result in a higher amount of variance explained by group separation and so the top 30 loadings of PC1 (Fig. 1S) are more closely related to metabolic differences across the sample cohort. This is in contrast to the AMIX models, which show variance explained mainly by outliers and their differences from the rest of the samples. AMIX PLS-DA models built for discriminating PA from PPGL samples all resulted in samples being incorrectly classified (Table 2S). In fact, the CPMG method resulted in the highest number of misclassified samples, probably because of outliers (Fig. 4b) that skew sample distribution and limit the performance of PLS-DA. On the other hand, the SPEAQ models resulted in no misclassifications. In addition, the optimum number of latent variables was usually lower in SPEAQ models, indicating group separation as the dominating source of variance. Differences in signature of differentiation based on the UF models (AMIX and SPEAQ) are summarized in Table 3S. Although a higher percentage of variables are VIPs for the AMIX dataset, more than half are integrals of regions with no detectable signals (noise variables). Also, there are buckets of regions containing only parts of peaks (split peaks), a phenomenon not possible with peak picking. Finally, more variables could be assigned to multiple metabolites in the AMIX than in the SPEAQ-derived signature. In light of these differences, we elected to continue with analyses of solely peak data for the comparison of sample preparation methods to each other.

Focusing on SPEAQ results, which led to a lower number of both latent variables and misclassifications (Table 2S), the differences based on the PLS-DA VIP list are summarized in Table 4S. LED resulted in PLS-DA-derived signatures, which had more peaks as well as metabolites in common with UF than CPMG did. We therefore concluded that the LED method is a more appropriate approach for large scale metabolomics and we assessed its quantification capabilities.

### LED quantification assessment

The initial experiment on 8 replicates samples was used to compare TSP to MA signals in filtered and unfiltered samples. The TSP signal in unfiltered samples had a lower intensity and had a much greater peak width than in filtered samples (11 Hz vs. 1 Hz, respectively). On the contrary, the MA peak had an peak width of ~ 1 Hz in filtered and unfiltered samples and in all samples the satellite peaks were visible (Fig. 2S). These results indicate MA does not bind to macromolecules (as opposed to TSP) and corroborate its use as an internal standard in unfiltered plasma NMR studies.

#### Linearity of Response

Results of the linearity assessment are summarized in Table 5S. All adjusted coefficients of determination (R^2^) were above 0.75 for all models, and significant, with a P value less than 0.05, indicative of a linear relationship.

#### LOD/LOQ

Metabolite LODs were all in the low micromolar range. All LOD and LOQ values for metabolites measured can be found in Table 5S.

#### Recovery

Recovery percentages can be found in Table 6S, for all metabolite quantities added in the standard addition experiments. Average recovery was higher than 65% for all metabolites except for creatinine. Furthermore, convoluted signals presented with observed recovery of above 100%, while, as a rule, lower concentrations were accompanied by lower recovery rates.

#### Agreement with amino acid assay

For threonine, a positive overall bias of 21 μM was found, with analyte range from − 13 to 47 μM, indicating limited agreement, as this bias is observed even at low concentrations. A negative bias of 4 μM was found for Methionine, and differences range from − 9 to 2 μM, indicating acceptable agreement. Glycine results seem to partially agree, since there is a negative average bias (indicating protein binding) of 20 μM and differences (− 106–65 μM) are low compared to the concentrations of this analyte. Lysine was quantified both using Chenomx and by directly integrating the peak at 3.01 ppm, but overall agreement was low in both cases, with NMR results being consistently lower than AAA when using Chenomx and higher after direct integration. Alanine presented with an average lack of bias, which ranged from − 116 to 82 μM and, just like glycine, small differences compared to this metabolite’s levels (Fig. 3S). The limited agreement found for lysine and threonine can be attributed to the high level of convolution their signals present with.

#### Repeatability

The inter-batch median RSD for all metabolites was found to be 10.8%, slightly lower than the 11.4% median intra-batch RSD. A total of 91 (85%) out of all 107 peaks detected had an inter-batch RSD < 30%, whereas 93 (87%) of all peaks had an intra-batch RSD below 30%. Highly variable peaks were either near the detection limit, high intensity or in close proximity to other peaks.

## Discussion

We describe here a comparison of methods for analyzing plasma samples with ^1^H-NMR spectroscopy for large studies, with the aim of separating groups of samples on the basis of untargeted metabolome differences. We compare the performance of the proposed approach to the commonly used CPMG method, on the basis of detected NMR signals and sample classification using multivariate statistics, using the UF as a gold standard for the comparison. For large studies, UF is not applicable but when comparing LED to CPMG spectra, an important result found was the increased number of detectable metabolites in LED spectra combined with a more efficient suppression of macromolecular signals. This is the first time that LED is shown, experimentally, not to suffer from these known (de Graaf and Behar 2003) limitations of the relaxation-editing CPMG method. In our current study, we demonstrate that, when applied in a simple sample classification experiment (separating PA from PPGL replicates) using supervised MVA, the metabolic signature of the LED model closely resembles the one derived from UF results, whereas the CPMG-derived list of VIPs gives rise to important differences with this gold standard. Even so, the definitive performance of our LED-based workflow on a large sample set with the goal of disease biomarker discovery remains to be assessed, as this analysis was done on a relatively limited sample cohort of replicates (18 in total, 9 per group). A method that has been demonstrated to be even more accurate especially in terms of metabolite quantitation involves methanol precipitation (Nagana Gowda and Raftery 2014), but was not selected as a gold standard due to residual macromolecule signals and the fact that it is mostly aimed at targeted studies, which are difficult to implement to large cohorts even in plasma, given variations in peak widths and chemical shifts that introduce artificial variance when automated (Bingol 2018).

We also present optimization results of the spectral processing pipeline. As described in the results section, a peak picking algorithm implemented in R package “SPEAQ” was selected after comparison with conventional binning. This is because, relative to spectral binning, the peak picking workflow used in our study leads to more accurate multivariate models for group separation by ignoring noise signals that, by definition, only offer information useful for analytical measurements. The workflow selected also results in more robust models that are less affected by technical outliers, at least based on our data. The VIP signature obtained by SPEAQ models, is also an advantage compared to AMIX. It is more closely related to metabolites (since less noise variables were found to be important) and interpretation is more direct, because a fewer number of peaks arise from multiple metabolites. These advantages are of particular importance for building supervised classification models since their accuracy (and thus their usefulness for making predictions) is dependent on the amount of variation explained that is relevant to the biological question (Westerhuis et al. 2010). Although there are newer and more efficient alternatives to binning (Emwas et al. 2018), the approach described is still used in recent publications (Castiglione Morelli et al. 2019; Hanifa et al. 2019; Jiang et al. 2019; Singh et al. 2019), while the method provided by the SPEAQ R package is an improvement, based on both our results presented here and the corresponding paper (Beirnaert et al. 2018). The absence of an alignment step can be explained by both the relatively stable chemical shift for peaks across samples, due to stable pH, explained by blood homeostasis and the use of a buffer solution. Furthermore, the SPEAQ peak picking procedure includes a peak grouping step which groups peaks with a slightly different chemical shift, as detected during peak picking (Beirnaert et al. 2018). Following peak picking and grouping, the workflow is made complete by a number of additional steps, which are all based on the protocol described by Southam et al. (2017). Given how peak picking is selected as a superior method to equidistant bucketing, this protocol and all its steps are made relevant to the data used in our study. Notable deviations from this workflow include the scaling of all detected peaks to the MA signal before PQN and the lack of batch correction. The use of the quantification reference for peak scaling was selected after comparison with the performance of total intensity scaling, which resulted in metabolite signal intensity values inconsistent with manual integration results (data not shown). Batch correction was not carried out, but any future applications of this method should include investigations of within- or between-batch effects. Intra-batch effects could be more likely, in fact, due to the absence of cooling on the carousel of the NMR spectrometer employed. Our results were found not to correlate with the order we analyzed our samples, due to proper randomization and an already high statistical power achieved when separating groups of replicate samples. However, it is recommended that the maximum number of samples per batch be estimated before implementation of the complete procedure to biomarker discovery studies.

We also introduce MA as an internal standard for untargeted metabolomics. As presented in this study, the MA peak at 6 ppm does not broaden in unfiltered compared to filtered samples, unlike TSP, the internal standard most frequently used. Even so, MA is a weak chemical shift axis calibration reference, owning to its singlet’s strong chemical shift dependency on pH. It is for this reason that plasma samples are calibrated to the glucose doublet at 5.22 ppm which is quite stable across samples and runs. Furthermore, the performance of MA in scaling NMR spectra has not been compared to that of other alternatives, such as the ERETIC virtual internal standard (Akoka et al. 1999; Albers et al. 2010), DSA (Alum et al. 2008) or formic acid (Beckonert et al. 2007).

To evaluate quantification, previous work (de Graaf and Behar 2003) compared diffusion-edited NMR to ultrafiltration and found excellent correlation between the two for most metabolites, with the exception of glycerol and citrate the levels of which were influenced by the use of filters. In our own work, quantification was assessed using the standard addition method and by comparison to a dedicated and validated analytical method for amino acids. A strong linear relationship was found based on the standard addition experiment for all metabolites investigated. Recovery results were mainly limited by signal convolution and by the inherent disadvantage of NMR methods in quantifying the bound fraction of a metabolite to protein. Method agreement was acceptable for most metabolites, but limited for lysine and threonine due to overlap with creatine and glycerol resonance signals, respectively. Although ultrafiltration is inferior to methanol precipitation in accurate plasma metabolite quantification according to the work of (Nagana Gowda and Raftery 2014), our results agree with theirs. Another consideration that may warrant investigation is the usage of pooled samples of the population under study in order to determine the appropriate correction factors as proposed by Wallmeier et al. (2017), although this would be applicable only to studies where ample research sample volume is available for QC samples. To evaluate repeatability, both inter- and intra-batch RSD of all peaks was calculated. Median RSD for inter- as well as intra-batch was found to be well below the FDA cutoff of 30% (Crews 2013) for 85–87% of all peaks. Nevertheless, 13–15% of the total number of peaks were found to have relatively high intra- and inter-batch RSD. These peaks were the result of peak picking errors, which can be explained by either low resolution due to a peak being near the detection limit, heteroscedasticity that results in high variance in high intensity peaks or the presence of nearby signals, which led to the algorithm used missing the peak in question in a subset of samples.

## Conclusions

In conclusion, here we present a comparison of ^1^H-NMR approaches for analyzing the human plasma metabolome, using a streamlined process for generating multivariate models. Although the traditional UF still detected the largest number of metabolites, LED is capable of detecting more metabolites and leads to PLS-DA models more similar to UF than CPMG, an important conclusion given that CPMG is the method of choice for large scale NMR metabolomics. Thus, we propose a facile new approach that was shown to be a viable alternative to the laborious and time-consuming conventional UF, more appropriate and cost-efficient for large-scale studies. Overall, our results suggest that the proposed approach is expected to yield valuable results in studies aimed at patient stratification and big data integration, and lead to new metabolites for detecting disease.

## Electronic supplementary material

Below is the link to the electronic supplementary material.

Supplementary file1 (DOCX 26 kb)

Supplementary file2 (TIFF 4433 kb)

Supplementary file3 (JPG 3548 kb)

Supplementary file4 (TIFF 4433 kb)

Supplementary file5 (DOCX 17 kb)

Supplementary file6 (DOCX 13 kb)

Supplementary file7 (DOCX 13 kb)

Supplementary file8 (DOCX 13 kb)

Supplementary file9 (DOCX 14 kb)

Supplementary file10 (DOCX 13 kb)

## Abbreviations

CPMG: Carr–Purcell–Meiboom–Gill
LED: Longitudinal Eddy-current delay
NMR: Nuclear magnetic resonance
TSP: Trimethylsilylpropanoic acid
MA: Maleic acid
UF: Ultrafiltration
PCA: Principal component analysis
PLS-DA: Partial least squares–Discriminant analysis
VIP: Variable importance to the projection
CV: Cross validation
NOESY: Nuclear overhauser effect spectroscopy
MVA: Multivariate analysis
RSD: Relative standard deviation (a.k.a. Coefficient of Variation)
