# Supplementary material for: A comparison of high-throughput plasma NMR protocols for comparative untargeted metabolomics
Source: Metabolomics. 2020 May 1;16(5):64. doi: 10.1007/s11306-020-01686-y (PMC7196944; doi:10.1007/s11306-020-01686-y)
Supplement: Supplementary file 1 — Supplementary file1 (DOCX 26 kb) [file 11306_2020_1686_MOESM1_ESM.docx]

A COMPARISON OF HIGH-THROUGHPUT PLASMA NMR PROTOCOLS FOR COMPARATIVE UNTARGETED METABOLOMICS

Nikolaos G. Bliziotis^1*^ · Udo F.H. Engelke^1^ · Ruud L.E.G. Aspers^2^ · Jasper Engel^2,3^ · Jaap Deinum^4^ · Henri J.L.M. Timmers^4^ · Ron A. Wevers^1^ · Leo A.J. Kluijtmans^1**^

^1^ Translational Metabolic Laboratory, Department of Laboratory Medicine, Radboudumc, Geert Grooteplein Zuid 10, 6525 GA Nijmegen, the Netherlands

^2^ Institute for Molecules and Materials, Radboud University, Houtlaan 4, 6525 XZ Nijmegen, the Netherlands

^3^ Current Address: Biometris, Wageningen UR, Droevendaalsesteeg 1, 6708, PB Wageningen, The Netherlands

^4^ Department of Internal Medicine, Radboudumc, Geert Grooteplein Zuid 10, 6525 GA Nijmegen, the Netherlands

Corresponding authors:

*Tel: +31 24 361 45 67. Fax: +31 24 366 87 54.

E-mail: nick.bliziotis@radboudumc.nl

**Tel: +31 24 361 45 67. Fax: +31 24 366 87 54.

E-mail: leo.kluijtmans@radboudumc.nl

Contents

[Figure 1S: Loadings plots of the Figure 4 PCA models, showing the top 30 contributing bins/peaks 2](#_Toc37248321)

[Table 1S: Detectable metabolites, their corresponding peaks and their presence in the PPGL spectra of each method. 2](#_Toc37248322)

[Table 2S: PLS-DA model characteristics after double cross validation. 2](#_Toc37248323)

[Table 3S: Comparison of PLS-DA-generated VIPs based on the AMIX and SPEAQ UF datasets. 2](#_Toc37248324)

[Table 4S: Comparison of PLS-DA-generated VIPs based on the UF, CPMG and LED SPEAQ datasets. 2](#_Toc37248325)

[Figure 2S: The maleic acid peak at 6 ppm compared to the TSP peak at 0 ppm in both filtered and unfiltered plasma samples. 2](#_Toc37248326)

[Table 5S: The results from linearity assessment for each of the metabolites spiked. 2](#_Toc37248327)

[Table 6S: Percentage of recovery (%) for each metabolite and each concentration level added in QC samples. 2](#_Toc37248328)

[Figure 3S: Bland-Altman plots of agreement for 5 metabolites between the two methods investigated 2](#_Toc37248329)

Figure 1S: Loadings plots of the Figure 4 PCA models, showing the top 30 contributing bins/peaks: (a) UF bucket table, (b) CPMG bucket table, (c) LED bucket table, (d) UF peak table, (e) CPMG peak table, and (f) LED peak table. The horizontal axes of all plots depict loading weights per variable in the PCA model, whereas the vertical axes display each variable (bin or peak). All 30 features have loading weights in only one direction in AMIX models, highlighting the peaks most important for separating outliers from the rest. On the other hand, in SPEAQ models loading weights alternate between directions and explain differences between groups.

# Table 1S: Detectable metabolites, their corresponding peaks and their presence in the PPGL spectra of each method. Macromolecules are not detectable in UF spectra, whereas lysine and ornithine are detectable in LED and not CPMG. Pubchem CID, along with the level of identification rigor is also reported, according to MSI guidelines. Metabolites with no identification level reported have only a singular peak in NMR spectra, a property which is insufficient for identification according to MSI guidelines.

# Table 2S: PLS-DA model characteristics after double cross validation. NMC: number of samples incorrectly classified after double cross validation out of a total of 18, LV: number of latent variables selected by performance assessment of the classification model.

# Table 3S: Comparison of PLS-DA-generated VIPs based on the AMIX and SPEAQ UF datasets. The total number of features obtained, along with the number of those with VIP>1, and those corresponding to part of a metabolite peak (split peaks), multiple metabolites and noise variables are listed, along with the number of corresponding metabolites.

# Table 4S: Comparison of PLS-DA-generated VIPs based on the UF, CPMG and LED SPEAQ datasets. The total number of features obtained from each method using SPEAQ, the number of those important for each PLS-DA model, the number of the VIPs also found in the UF PLS-DA model (common important peaks), the total number of assignable metabolites based on the VIPs found and the metabolites that were also found important via the PLS-DA model.

# Figure 2S: The maleic acid peak at 6 ppm compared to the TSP peak at 0 ppm in both filtered and unfiltered plasma samples. The maleic acid peak is unaffected by the presence of protein and does not result in peak broadening in unfiltered samples. Both the singlet and the peak satellites can be observed.

# Table 5S: The results from linearity assessment for each of the metabolites spiked. Columns contain results from each linear model, specifically on the coefficient of determination (R2) with its associated p-value, standard error for the regression (RSE), as well as estimated limits of detection (LOD) and quantitation (LOQ).

# Table 6S: Percentage of recovery (%) for each metabolite and each concentration level added in QC samples. Last column depicts the average recovery of all concentration levels.

Figure 3S: Bland-Altman plots of agreement for 5 metabolites between the two methods investigated (proposed LED approach vs. amino acid assay). (a) Threonine, (b) Methionine, (c) Glycine, (d) Lysine, (e) Alanine. All concentration estimates that were used as input for the plots were expressed in mM.
