# Supplementary figures and images for: A comparison of high-throughput plasma NMR protocols for comparative untargeted metabolomics
Source: Metabolomics. 2020 May 1;16(5):64. doi: 10.1007/s11306-020-01686-y (PMC7196944; doi:10.1007/s11306-020-01686-y)

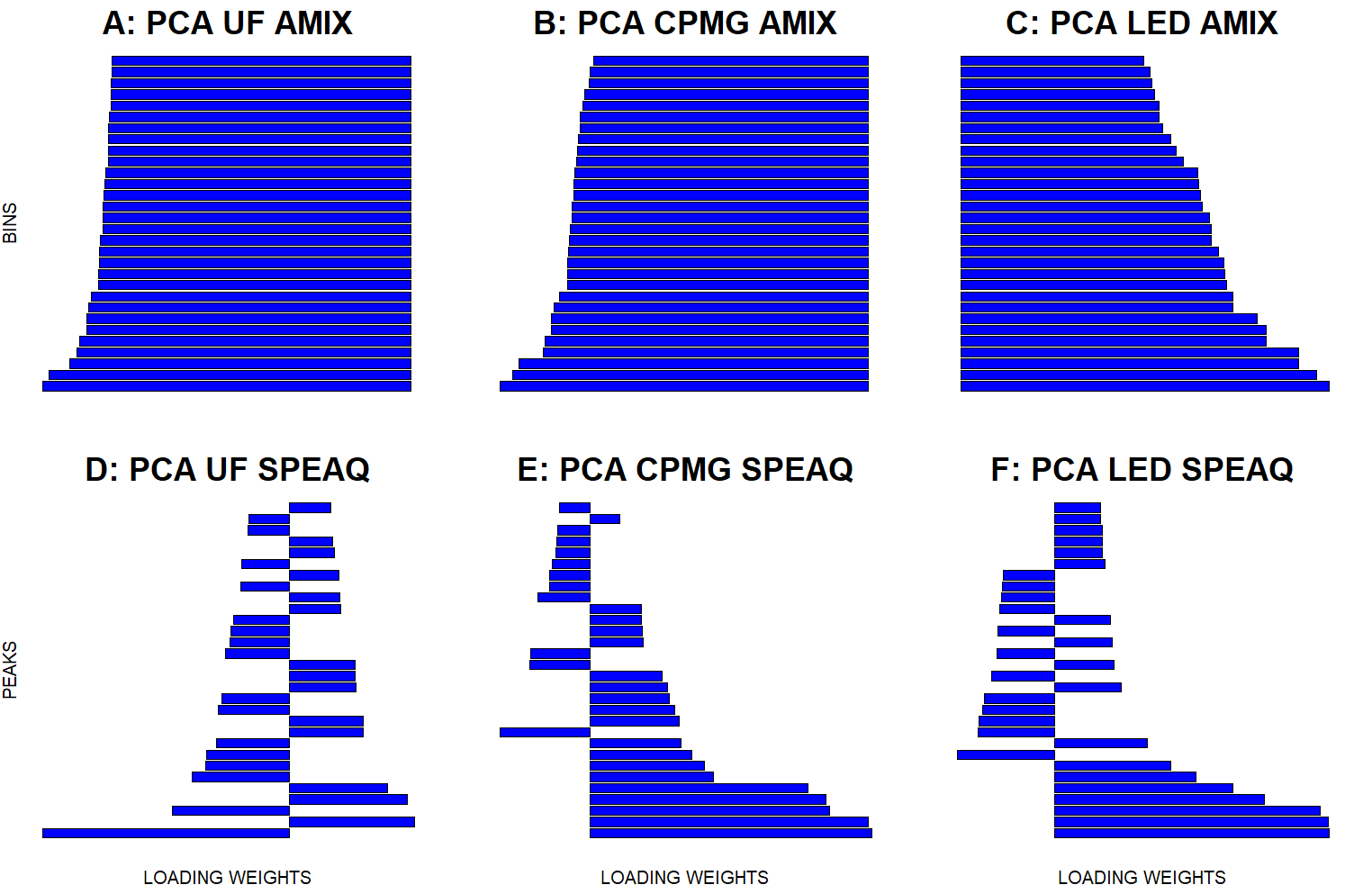

Supplement: Supplementary file 2 — Supplementary file2 (TIFF 4433 kb) [file 11306_2020_1686_MOESM2_ESM.tiff]

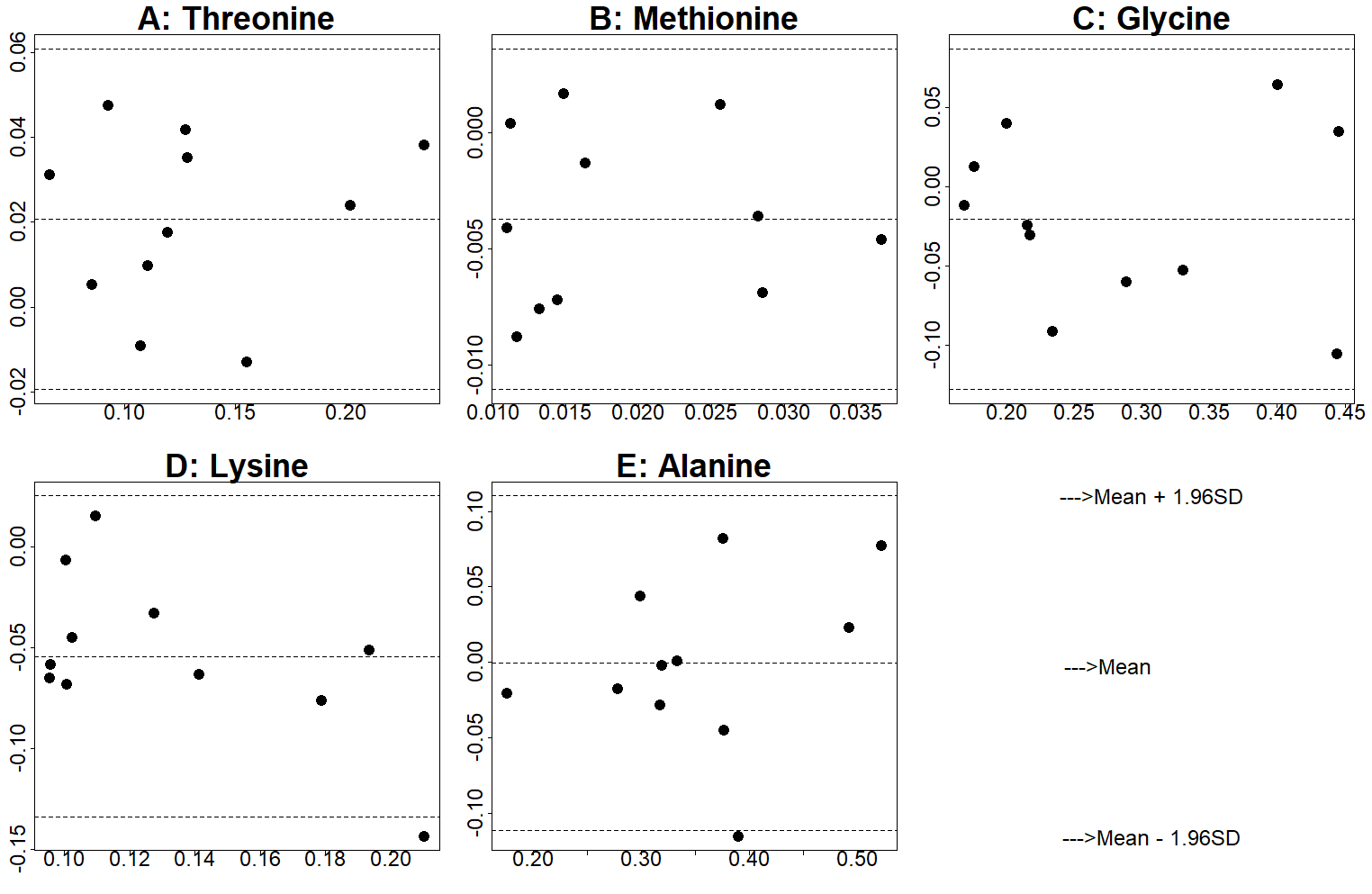

Supplement: Supplementary file 4 — Supplementary file4 (TIFF 4433 kb) [file 11306_2020_1686_MOESM4_ESM.tiff]
