# Supplementary material for: A comparison of high-throughput plasma NMR protocols for comparative untargeted metabolomics
Source: Metabolomics. 2020 May 1;16(5):64. doi: 10.1007/s11306-020-01686-y (PMC7196944; doi:10.1007/s11306-020-01686-y)
Supplement: Supplementary file 5 — Supplementary file5 (DOCX 17 kb) [file 11306_2020_1686_MOESM5_ESM.docx]

| Metabolite | Peaks (ppm) | CID | Level of  Identification rigor | Detected in PPGL UF spectrum | Detected in PPGL CPMG spectrum | Detected in PPGL LED spectrum |
| --- | --- | --- | --- | --- | --- | --- |
| Formate | 8.44 s | 283 |  | + | + | + |
| Histidine (His) | 7.8 s, 7.06 s | 6274 | 2 | + | + | + |
| Phenylalanine (Phe) | 7.42 m, 7.36 m, 7.31 m | 6140 | 2 | + | - | - |
| Tyrosine (Tyr) | 7.18 m, 6.89 m | 6057 | 2 | + | + | + |
| Urea | 5.78 broad | 1176 |  | + | - | - |
| Glucose | 5.22 d, 4.63 d, 3.89 dd, 3.82 m, 3.76 dd, 3.72 dd, 3.70 t, 3.52 dd, 3.48 t, 3.45 m, 3.40 t, 3.39 t, 3.23 dd | 5793 | 2 | + | + | + |
| Threonine (Thr) | 4.24 m, 3.57 d | 6288 | 2 | + | + | + |
| Proline (Pro) | 4.12 dd, 3.32 m, 2.34 m, 2.05 m, 2.02 m, 1.97 m | 614 | 2 | + | + | + |
| Lactate | 4.10 q, 1.31 d | 612 | 2 | + | + | + |
| Creatinine | 4.04 s, 3.03 s | 588 | 2 | + | + | + |
| Serine (Ser) | 3.98 dd, 3.93 dd | 5951 | 2 | + | + | + |
| Creatine | 3.92 s, 3.02 s | 586 | 2 | + | + | + |
| Isoleucine (Ile) | 3.66 d, 0.99 d, 0.92 t | 6306 | 2 | + | + | + |
| Glycerol | 3.64 dd, 3.55 dd | 753 | 2 | + | + | + |
| Valine (Val) | 3.60 d, 2.26 m, 1.03 d, 0.97 d | 6287 | 2 | + | + | + |
| Glycine (Gly) | 3.55 s | 750 | 2 | + | + | + |
| Methanol | 3.35 s | 887 |  | + | + | + |
| Ornithine | 3.04 t, 1.94 m | 6262 | 2 | + | - | + |
| Lysine (Lys) | 3.01 t, 1.91 m, 1.87 m, 1.71 m | 5962 | 2 | + | - | + |
| Asparagine (Asn) | 2.94 dd, 2.84 dd | 6267 | 2 | + | - | - |
| Dimethylglycine | 2.91 s | 673 |  | + | + | + |
| Dimethylamine | 2.70 s | 674 |  | + | + | + |
| Citrate | 2.66 d, 2.52 d | 311 | 2 | + | + | + |
| Methionine (Met) | 2.63 t, 2.12 s | 6137 | 2 | + | + | + |
| 2-Oxoisocaproate | 2.60 d, 0.92 d | 70 | 2 | + | - | - |
| Glutamine (Gln) | 2.44 m, 2.12 m | 5961 | 2 | + | + | + |
| Pyruvate | 2.36 s | 107735 |  | + | + | + |
| Acetoacetate | 2.26 s | 6971017 |  | + | + | + |
| Acetone | 2.21 s | 180 |  | + | + | + |
| Fatty acyl chain CH_2_CH= | 2.02 broad |  |  | - | + | + |
| Acetate | 1.90 s | 175 |  | + | + | + |
| Alanine (Ala) | 1.46 d | 5950 | 2 | + | + | + |
| Fatty acyl chain methylene | 1.26 broad |  |  | - | + | + |
| 3-hydroxybutyrate | 1.19 d | 3541112 | 2 | + | - | - |
| 3-methyl-2-oxovalerate | 1.11 d | 47 | 2 | + | - | - |
| Isobutyrate | 1.06 d | 165337 | 2 | + | - | - |
| Leucine (Leu) | 1.7 m, 0.95 t | 6106 | 2 | + | + | + |
| Fatty acyl chain methyl | 0.87 broad |  |  | - | + | + |
| 2-hydroxybutyrate | 0.88 t | 4071895 | 2 | + | - | - |
