# Supplementary material for: A comparison of high-throughput plasma NMR protocols for comparative untargeted metabolomics
Source: Metabolomics. 2020 May 1;16(5):64. doi: 10.1007/s11306-020-01686-y (PMC7196944; doi:10.1007/s11306-020-01686-y)
Supplement: Supplementary file 6 — Supplementary file6 (DOCX 13 kb) [file 11306_2020_1686_MOESM6_ESM.docx]

|  | AMIX UF | AMIX CPMG | AMIX LED | SPEAQ UF | SPEAQ CPMG | SPEAQ LED |
| --- | --- | --- | --- | --- | --- | --- |
| NMC | 1 (6%) | 8 (44%) | 1 (6%) | 0 (0%) | 0 (0%) | 0 (0%) |
| LV | 3 | 7 | 2 | 1 | 2 | 2 |
