# Supplementary material for: A comparison of high-throughput plasma NMR protocols for comparative untargeted metabolomics
Source: Metabolomics. 2020 May 1;16(5):64. doi: 10.1007/s11306-020-01686-y (PMC7196944; doi:10.1007/s11306-020-01686-y)
Supplement: Supplementary file 7 — Supplementary file7 (DOCX 13 kb) [file 11306_2020_1686_MOESM7_ESM.docx]

|  | Total  bins/peaks | VIPs | Split Peaks | Variables with multiple metabolites | Noise variables | Important known metabolites |
| --- | --- | --- | --- | --- | --- | --- |
| AMIX | 381 | 100 (26%) | 23 | 9 | 54 | 16 |
| SPEAQ | 378 | 74 (20%) | 0 | 4 | 3 | 17 |
