# Supplementary material for: A comparison of high-throughput plasma NMR protocols for comparative untargeted metabolomics
Source: Metabolomics. 2020 May 1;16(5):64. doi: 10.1007/s11306-020-01686-y (PMC7196944; doi:10.1007/s11306-020-01686-y)
Supplement: Supplementary file 8 — Supplementary file8 (DOCX 13 kb) [file 11306_2020_1686_MOESM8_ESM.docx]

|  | Total features | Total VIP | Common VIP | Important known Metabolites | Common known Metabolites |
| --- | --- | --- | --- | --- | --- |
| UF | 378 | 74 (20%) | 74 (100%) | 17 | 17 |
| CPMG | 118 | 30 (25%) | 16 (22%) | 7 | 7 |
| LED | 122 | 25 (20%) | 19 (26%) | 9 | 8 |
