# Supplementary material for: A comparison of high-throughput plasma NMR protocols for comparative untargeted metabolomics
Source: Metabolomics. 2020 May 1;16(5):64. doi: 10.1007/s11306-020-01686-y (PMC7196944; doi:10.1007/s11306-020-01686-y)
Supplement: Supplementary file 9 — Supplementary file9 (DOCX 14 kb) [file 11306_2020_1686_MOESM9_ESM.docx]

| **MODEL** | **Adjusted R^2^** | **P value** | **RSE** | **Prediction Error %** | **LOD**  **(μM)** | **LOQ**  **(μM)** |
| --- | --- | --- | --- | --- | --- | --- |
| **GLYCINE** | 0.8544 | 5.19E-07 | 0.1153 | 23.57529 | 20 | 70 |
| **ALANINE** | 0.7741 | 9.36E-06 | 0.04989 | 12.43789 | 14 | 46 |
| **PHENYLALANINE** | 0.99 | 1.35E-14 | 0.02713 | 6.806533 | 10 | 30 |
| **METHIONINE** | 0.9538 | 2.84E-10 | 0.004976 | 13.31305 | 2.2 | 7.3 |
| **LYSINE** | 0.9828 | 4.54E-13 | 0.0218 | 7.554596 | 7 | 23 |
| **THREONINE** | 0.7259 | 3.37E-05 | 0.02558 | 15.16965 | 10 | 30 |
| **CREATINE** | 0.9298 | 4.37E-09 | 0.01096 | 13.83609 | 5 | 17 |
| **CREATININE** | 0.8885 | 8.99E-08 | 0.009616 | 14.41108 | 4.8 | 15.8 |
