# Supplementary material for: A comparison of high-throughput plasma NMR protocols for comparative untargeted metabolomics
Source: Metabolomics. 2020 May 1;16(5):64. doi: 10.1007/s11306-020-01686-y (PMC7196944; doi:10.1007/s11306-020-01686-y)
Supplement: Supplementary file 10 — Supplementary file10 (DOCX 13 kb) [file 11306_2020_1686_MOESM10_ESM.docx]

| **METABOLITE** | **Concentration 1 (Highest)** | **Concentration 2** | **Concentration 3** | **Concentration 4 (Lowest)** | **Average** |
| --- | --- | --- | --- | --- | --- |
| **GLYCINE** | 72 | 99.7 | 118 | 97 | 96.68 |
| **ALANINE** | 57 | 76 | 121 | 70 | 81 |
| **PHENYLALANINE** | 78 | 73 | 90 | 89 | 82.5 |
| **METHIONINE** | 56 | 69 | 73 | 62 | 65 |
| **LYSINE** | 71 | 67 | 93 | 60 | 72.75 |
| **THREONINE** | 53 | 85 | 122 | 39 | 74.75 |
| **CREATINE** | 53 | 67 | 83 | 71 | 68.5 |
| **CREATININE** | 39 | 46 | 66 | 40 | 47.75 |
